# CRH-R2 signalling modulates feeding and circadian gene expression in hypothalamic mHypoA-2/30 neurons

**DOI:** 10.3389/fendo.2023.1266081

**Published:** 2023-10-11

**Authors:** Viridiana Alcántara-Alonso, Robert Dallmann, Hendrik Lehnert, Patricia de Gortari, Dimitris K. Grammatopoulos

**Affiliations:** ^1^ Translational Medicine, Warwick Medical School, University of Warwick, Coventry, United Kingdom; ^2^ Laboratorio de Neurofisiología Molecular, Instituto Nacional de Psiquiatría Ramón de la Fuente Muñiz, Ciudad de México, Mexico; ^3^ Rectorate, Paris Lodron Universität Salzburg, Salzburg, Austria; ^4^ Institute of Precision Diagnostics and Translational Medicine, Pathology, University Hospital Coventry and Warwickshire (UHCW), National Health Service (NHS) Trust, Coventry, United Kingdom

**Keywords:** Feeding, hypothalamic peptides, CRH-R2, urocortin 2 (UCN2), AMPK, CREB, circadian rhythm

## Abstract

The hypothalamic type 2 corticotropin releasing hormone receptor (CRH-R2) plays critical roles in homeostatic regulation, particularly in fine tuning stress recovery. During acute stress, the CRH-R2 ligands CRH and urocortins promote adaptive responses and feeding inhibition. However, in rodent models of chronic stress, over-exposure of hypothalamic CRH-R2 to its cognate agonists is associated with urocortin 2 (Ucn2) resistance; attenuated cAMP-response element binding protein (CREB) phosphorylation and increased food intake. The molecular mechanisms involved in these altered CRH-R2 signalling responses are not well described. In the present study, we used the adult mouse hypothalamus-derived cell line mHypoA-2/30 to investigate CRH-R2 signalling characteristics focusing on gene expression of molecules involved in feeding and circadian regulation given the role of clock genes in metabolic control. We identified functional CRH-R2 receptors expressed in mHypoA-2/30 cells that differentially regulate CREB and AMP-activated protein kinase (AMPK) phosphorylation and downstream expression of the appetite-regulatory genes proopiomelanocortin (*Pomc*) and neuropeptide Y (*Npy*) in accordance with an anorexigenic effect. We studied for the first time the effects of Ucn2 on clock genes in native and in a circadian bioluminescence reporter expressing mHypoA-2/30 cells, detecting enhancing effects of Ucn2 on mRNA levels and rhythm amplitude of the circadian regulator Aryl hydrocarbon receptor nuclear translocator-like protein 1 (*Bmal1*), which could facilitate anorexic responses in the activity circadian phase. These data uncover novel aspects of CRH-R2 hypothalamic signalling that might be important in regulation of circadian feeding during stress responses.

## Introduction

Feeding behaviour in mammals is modulated by an array of stimuli and hormonal signals that converge in the hypothalamus where the information about mood, energy status and time-of-day (light/dark period) is processed in order to initiate the search for food or conversely to stop eating; aligned with the activity/resting phases, respectively.

It is clearly established that physiological, biochemical and behavioural responses to stress involve regulation of feeding behaviour and energy balance (1). Stressful stimuli can exert divergent effects on appetite depending on the intensity, type and duration of the stressor. Exposure to acute stress induces release of corticotropin releasing hormone (CRH) and urocortins 1-3 (Ucn1, Ucn2 and Ucn3) from hypothalamic cells, leading to elevated glucocorticoids serum concentration and suppression of appetite and feeding. In contrast, during a chronic stress exposure, energy reservoirs are utilized and depleted by glucocorticoids action; thus, a compensatory hyperphagia may develop to replenish energy stores (2).

CRH and urocortins exert acute anorexigenic effects via binding to type 2 CRH receptors (CRH-R2) in the hypothalamus (1). Interestingly, our previous study in a rodent model of chronic early-life stress suggested that over-exposure of hypothalamic CRH-R2 to its cognate agonists (CRH, Ucn2) induces Ucn2 resistance, along with attenuated CREB phosphorylation and increased food intake in adulthood (3) however, the molecular pathways involved in this maladaptive functional response are poorly understood.

CRH-R2 is a G protein coupled receptor (GPCR) (4) highly expressed in the ventromedial, arcuate and paraventricular hypothalamic nuclei (5). In most tissues including the brain, its intracellular signalling is primarily but not exclusively mediated via activation of the Gα_s_ protein, promoting increases in intracellular cyclic AMP (cAMP) levels and protein kinase A (PKA) activity (6). One of the downstream targets of PKA is the transcription factor cAMP response element binding protein (CREB); its phosphorylation at Ser133 induces its binding to the CRE region of target promoters and the initiation of gene transcription (7).

Importantly, feeding-regulatory peptides synthesized in the hypothalamic arcuate nucleus such as Neuropeptide Y (*Npy*), Agouti related protein (*Agrp*) and proopiomelanocortin (*Pomc*) contain CRE binding sites in their promoters, thus acute or sustained elevated concentration of Ucn2 might differentially regulate appetite by changing the CRH-R2 transducing properties (8–12). However, modulation of these hypothalamic appetite-related genes by CREB is complex and is dependent on multiple hormonal inputs also from ghrelin, leptin or insulin (13).

Furthermore, another signalling molecule implicated in the transcription of these feeding-related peptides in the arcuate nucleus is the energy sensor AMP-activated protein kinase (AMPK), which is activated allosterically by a rise in AMP : ATP ratio and phosphorylation of the Thr172 residue of its α-subunit by calcium/calmodulin dependent protein kinase kinase (CaMKK2) or by liver kinase B1 (LKB1). Increased activity of AMPK in the hypothalamus increases food intake by promoting orexigenic neuropeptide expression including AgRP, NPY, orexins and melanin-concentrating hormone (MCH), while suppressing anorexigenic signals such as POMC or cocaine and amphetamine-regulated transcript (CART) (14). In contrast, AMPK inhibition induces hypophagia (15, 16). Hypothalamic AMPK activity is stimulated by ghrelin to promote feeding behaviour and inhibited by the anorectic hormones insulin or leptin (13).

Alongside hormonal signals, the suprachiasmatic hypothalamic nucleus (SCN) also receives light-related inputs from the retina and controls food intake in a circadian fashion (17), promoting eating during the activity phase of the animal, which is the light phase of the day for humans and the dark phase for most of the experimental laboratory rodents. Disturbances in the normal light/dark cycle are thought to promote hyperphagia through alterations in hypothalamic neuroendocrine systems (18). Stress exposure and sustained high Ucn2 concentration in the brain may be involved in appetite changes due to alterations in the expression of circadian patterns-dependent clock genes in the hypothalamus (19), which may disrupt diurnal rhythms in hormonal release and consequently modifying appetite (20). For example, chronic stress significantly delays the acrophase of the Circadian Locomotor Output Cycles Kaput (*Clock)* and Aryl hydrocarbon receptor nuclear translocator-like protein 1 (*Bmal1)* genes expression in the SCN (21). The effects of direct hypothalamic CRH-R2 stimulation on clock genes have not been explored yet, nevertheless, intracerebroventricular (i.c.v) injection of Ucn2 promotes anorexigenic effects in the dark phase of the cycle (22) and mice with CRH-R2 knockdown in the ventromedial hypothalamus (VMH) by small hairpin RNA show increased food-intake also during the dark phase of the day (23), suggesting a CRH-R2 mediated effect on circadian oscillations potentially influencing eating behaviour.

Since there is a paucity of *in vitro* studies investigating CRH-R2 biology in cellular models of hypothalamic neurons, here we explored CRH-R2 signalling characteristics *in vitro* using the adult mice hypothalamic derived cell line, mHypoA-2/30. The functional coupling of CRH-R2 to key signalling mediators such as CREB and AMPK involved in feeding behaviour led us to investigate a potential role of CRH-R2 in the transcription of orexigenic and anorexigenic signals, as well as on the circadian core oscillator in these hypothalamic cells.

## Materials and methods

### Cell culture

The adult mouse derived hypothalamic neuronal cell line mHypoA-2/30 (CELLutions Biosystems Inc, Toronto, Canada), was used as an experimental model. These cells possess neuron-specific markers and function as effective models to study neuroendocrine signalling at the cellular level (24). Cells were grown in 6 well plates (Corning life Sciences, Flintshire, UK) with high glucose (HG) Dulbecco’s Modified Eagle’s Medium (DMEM, 4500 mg/L, Sigma-Aldrich, Dorset, UK) supplemented with 5% fetal bovine serum (FBS, Gibco, Paisley UK) and 1% penicillin-streptomycin (Biochrome AG, Berlin, Germany) maintained at 37°C and 5% CO2 until 90% confluency. A HG medium was used to reduce basal AMPK activity. In these HG conditions we explored effects of Ucn2 and AICAR on phospho-AMPK by Western Blot.

### Immunofluorescent confocal microscopy

Cells were seeded in 22 mm sterile coverslips treated with 10% poly-D-lysine (Sigma-Aldrich) in growth medium and incubated overnight to allow attachment to the coverslip.

The following day, cells were treated with Ucn2 (100 nM, Bachem, St Helens, UK) for 1 or 2 hours before been washed with phosphate buffered saline (PBS) and fixed with 4% paraformaldehyde (PFA, Sigma-Aldrich) for 30 min at room temperature. After washing with PBS, non-specific binding was blocked with 3% bovine serum albumin (BSA, Sigma-Aldrich) for 1 hr at room temperature. Coverslips were then washed with PBS and incubated overnight at 4°C with the primary antibodies mapping the extracellular domain of e-cadherin (sc-7870 rabbit, Santa Cruz Biotechnology, Santa Cruz California, USA) and CRH-R2 (sc-20550 goat, Santa Cruz Biotechnology) in a 1% BSA solution. The next day, the coverslips were washed with PBS and incubated 1 hr in the dark at room temperature with the secondary antibodies: goat anti-rabbit conjugated to Texas red (ab6719 Abcam, Cambridge, UK) and donkey anti-goat conjugated to fluorescein (FITC, sc-2024, Santa Cruz Biotechnology) in 1% BSA before been washed with PBS and treated again with 4% PFA for 10 min. Coverslips were then washed with PBS, and incubated with DAPI (D1306 ThermoFisher Scientific, Cambridge, UK) for 10 min at room temperature. Then, coverslips were washed and mounted in slides with DPX (Thermo Fisher Scientific).

The slides were examined under an oil immersion objective (60 X) using a Perkin Elmer UltraVIEU VoX spinning disk confocal microscope system. Images were acquired with the Velocity software (Perkin Elmer, Waltham MA, USA) and the fluorescence profiles were analysed using the bio-formats and colocalization colormap plugins from ImageJ software (https://imagej.net/Fiji, National Institutes of Health, Bethesda MD, USA).

### Immunoblotting

CRH-R2 intracellular signalling was studied by treating mHypoA-2/30 cells in 6 well plates with Ucn2 (100 nM) for various intervals (20-120 min). In another set of experiments, the cells were also treated with DMSO or the AMPK activator *N* ^1^-(β-D-Ribofuranosyl)-5-aminoimidazole-4-carboxamide (AICAR, 1 mM, Tocris Biocience, Bristol, UK) for 120 min.

After medium removal and washing with PBS, RIPA lysis buffer (Abcam) was used with protease and phosphatase inhibitors (Thermo Fisher Scientific) to extract proteins. Buffer and cell lysates were collected and centrifuged at 10,400 g for 15 min. Supernatants were collected and a 10 µl aliquot was used to determine protein concentration using the Pierce BSA protein assay kit (Thermo Fisher Scientific). The rest of the sample was diluted with the same volume of 2X Laemmli buffer (Sigma-Aldrich) and protein denatured at 95°C for 5 min.

Samples containing 20-30 µg of protein were loaded in 10% SDS-PAGE gels for electrophoresis and then transferred to nitrocellulose membranes (Amersham, GE healthcare Little Chalfont, UK). Membranes were then incubated with blocking solution (GE healthcare) for 1 hr and left overnight in solution containing primary antibodies for either CRH-R2 (48KDa, ab203585 Abcam), phospho-CREB (Ser 133, 43 kDa, 9198 cell signalling technology, Hampshire, UK), total CREB (43 kDa, 4820 cell signalling technology), phospho-AMPK (Thr 172, 62 kDa, 2535 cell signalling technology), total AMPK (62 kDa, 2532 cell signalling technology), phospho-Acetyl-CoA Carboxylase (phospho-ACC Ser 79, 280 kDa, 3661 cell signalling technology), total ACC (280 kDa, 3662 cell signalling technology) or β-Actin (45 kDa, 4970 cell signalling technology) all raised in rabbit and diluted in 3% BSA. After washings with Tris buffered saline 0.1% tween, membranes were then incubated for 1 hour with the secondary antibody (anti-rabbit DyLight 800 4X PEG conjugate, 5151 cell signalling technology) in 3% BSA for 1 hour. Protein bands were visualized with the Odyssey infrared imaging system (LICOR Biosciences, Cambridge, UK). Membranes were washed with stripping solution [25 mM glycine, 1% SDS (Sigma-Aldrich), pH 2] after the phosphorylated protein detection and then re-probed for the total protein imaging. The NIH ImageJ software was used for the analysis and quantification of fluorescent signals.

### Real-time quantitative polymerase chain reaction

Ucn2 mediated gene expression changes on *Crhr2*, feeding-related peptides and proteins involved in circadian rhythmicity, was evaluated in a series of experiments. Firstly, mHypoA-2/30 cells were treated with water or 100 nM Ucn2 for 24 hours to evaluate changes on *Crhr2* gene expression. Total RNA was extracted following the instructions of the GenElute mammalian total RNA miniprep kit (Sigma Aldrich). In some experiments, hypothalamic cells were pre-treated for 15 min with 1 µM antisauvagine 30 (ASG30, Tocris), a CRH-R2 antagonist, or water before treatment for 24 hours with 100 nM of Ucn2 or water. Total mRNA was extracted, and the effect of ASG30 on Ucn2-induced expression of the anorexigenic *Pomc*, neurotensin (*Nts)*, and orexigenic *Npy* and *Agrp* genes was determined.

In a different set of experiments, cells were stimulated with 100 nM of Ucn2 for 24 hours with or without a 1 hour pre-treatment step to induce CRH-R2 internalization. Total mRNA was extracted, and changes on *Pomc*, Nts, *Npy* and *Agrp* gene expression were analysed.

Finally, to study the effects of Ucn2 on the expression of the circadian genes *Clock*, Cryptochrome 1 and 2 (*Cry1, Cry2) and Bmal1*, mHypoA-2/30 cells were serum starved for 12 hours before adding 30% FBS medium (FBS shock) to synchronize the cells (25). After 30 min, 30% FBS medium was replaced with 5% FBS medium and cells were incubated with or without Ucn2 (100 nM) for different time intervals over a 24 hours period. Total RNA from the samples was then extracted.

The quality and quantity of total RNA was determined using a NanoDrop spectrophotometer (Thermo Fisher Scientific). To eliminate DNA contamination, the DNase I Amplification grade kit (Invitrogen, Paisley UK) was used in samples for 10 min at 65°C and cDNA synthetized using the High-Capacity RNA to cDNA kit (Applied Biosystems, Warrington Cheshire, UK) for 1 hour at 37°C.

PCR reactions targeting the low expression genes: *Pomc* and *Npy* were done using Taqman gene expression primers, Mm00435874_m1 (*Pomc*), Mm00445771_m1 (*Npy*) and housekeeping gene β-Actin Mm00607939_s1 (*Actb*) with the Fast Advanced Master-Mix (Thermo Fisher Scientific).

Expression of other genes of interest was evaluated using the Sensi FAST SYBR Lo-ROX kit reagents (Bioline, London, UK) and the following primer sets (**Table 1**):

**Table 1:** Sequence-specific primers used for qRT-PCR reactions.

Gene	Forward Primer (5’ -> 3’)	Reverse Primer (5’ -> 3’)
β-Actin *(Actb)*	GGCTGTATTCCCCTCCATCG	CCAGTTGGTAACAATGCCATGT
*Agrp*	AGAGTTCCCAGGTCTAAGTCTG	GCGGTTCTGTGGATCTAGCA
*Bmal1*	ACCTCGCAGAATGTCACAGGCA	CTGAACCATCGACTTCGTAGCG
*Clock*	GGCTGAAAGACGGCGAGAACTT	GTGCTTCCTTGAGACTCACTGTG
*Crhr2*	CATCCACCACGTCCGAGAC	CTCGCCAGGATTGACAAAGAA
*Cry1*	GGTTGCCTGTTTCCTGACTCGT	GACAGCCACATCCAACTTCCAG
*Cry2*	GGACAAGCACTTGGAACGGAAG	ACAAGTCCCACAGGCGGTAGTA
Neurotensin *(Nts)*	GCAAGTCCTCCGTCTTGGAAA	TGCCAACAAGGTCGTCATCAT
TATA Box Binding protein (*Tbp*)	GTGCCAGATACATTCCGCCT	CAAGCTGCGTTTTTGTGCAG

The Applied Biosystems 7500 Fast system was used, and the quantified mRNA levels were normalized using the ΔΔCt method: with the threshold cycle (Ct), ΔCt= Ct target – Ct housekeeping gene. The fold change of mRNA expression calculated by the formula: 2 ^-(ΔCt of experimental compound – ΔCt of vehicle)^.

### Circadian luciferase reporter assay

Lenti-viral particles of the circadian bioluminescence reporter constructs pLV7-Bsd-p(*Bmal1*)-dLuc (*Bmal1*-*luc*) a kind gift of Andrew C. Liu (26), were transfected with TransIT-LT (MIR 2300, Mirus-Bio, WI, USA, 2019) in HEK-293FT cells (Thermo Fisher Scientific) following the manufacturer’s protocol. The transfection control used was pWPI plasmid (Addgene, MA, USA). For target cell transduction with lenti-viral particles, a 10 cm dish of 50% confluent mHypoA-2/30 cells were incubated with 1ml filtered viral supernatant in the presence of 8 µg/ml polybrene (Sigma-Aldrich) for 6 hours. Three days after viral transduction, selection of infected cells was started by adding Blasticidin (5 µg/ml, Thermo Fisher Scientific) to the culture medium for 1 week.

Selected mHypoA2/30-*Bmal1*-*luc* cells were seeded in 35 mm dishes and at 50% confluency, cells were synchronised with 100nM dexamethasone (Sigma-Aldrich) for 30 min before washing with PBS and addition of 100 nM luciferin (Promega, Chilworth, UK) in phenol-free media as described elsewhere (27).

A LumiCycle 32 (Actimetrics, IL, US) was used for bioluminescence recordings at 37°C with 5% CO_2_. The bioluminescence signal was recorded every 10 min. After 36 hours, mHypoA2/30- *Bmal1*-*luc* cells were treated with Ucn2 (100 nM) or water and the recording continued for another 4 days. The LumiCycle Analysis program was used to obtain baseline-subtracted data (27).

### Statistical analysis

Changes in colocalization of CRH-R2 and e-cadherin as well as phosphorylation of second messengers at different time points and gene expression of feeding peptides with 2 stimulations of Ucn2 were analysed by one-way ANOVA. Results of phosphorylation by AICAR or Ucn2 *vs* vehicles, gene and protein expression of *Crhr2*, as well as circadian modulators were analysed by Student’s *t* test. The circadian period was determined by chi square-periodogram analysis and the statistical significance of each of the variables were also analysed by Student’s *t* test. Comparison between gene expression in cells treated with Ucn2 with or without pre-treatment with ASG30 was carried out by two-way ANOVA and Fisher’s *post hoc* test performed using the Stat-View 5 software (SAS Institute Cary, NC, USA).

## Results

### CRH-R2 endogenous expression and signalling characteristics in mHypoA-2/30 cells

**Figure 1:**
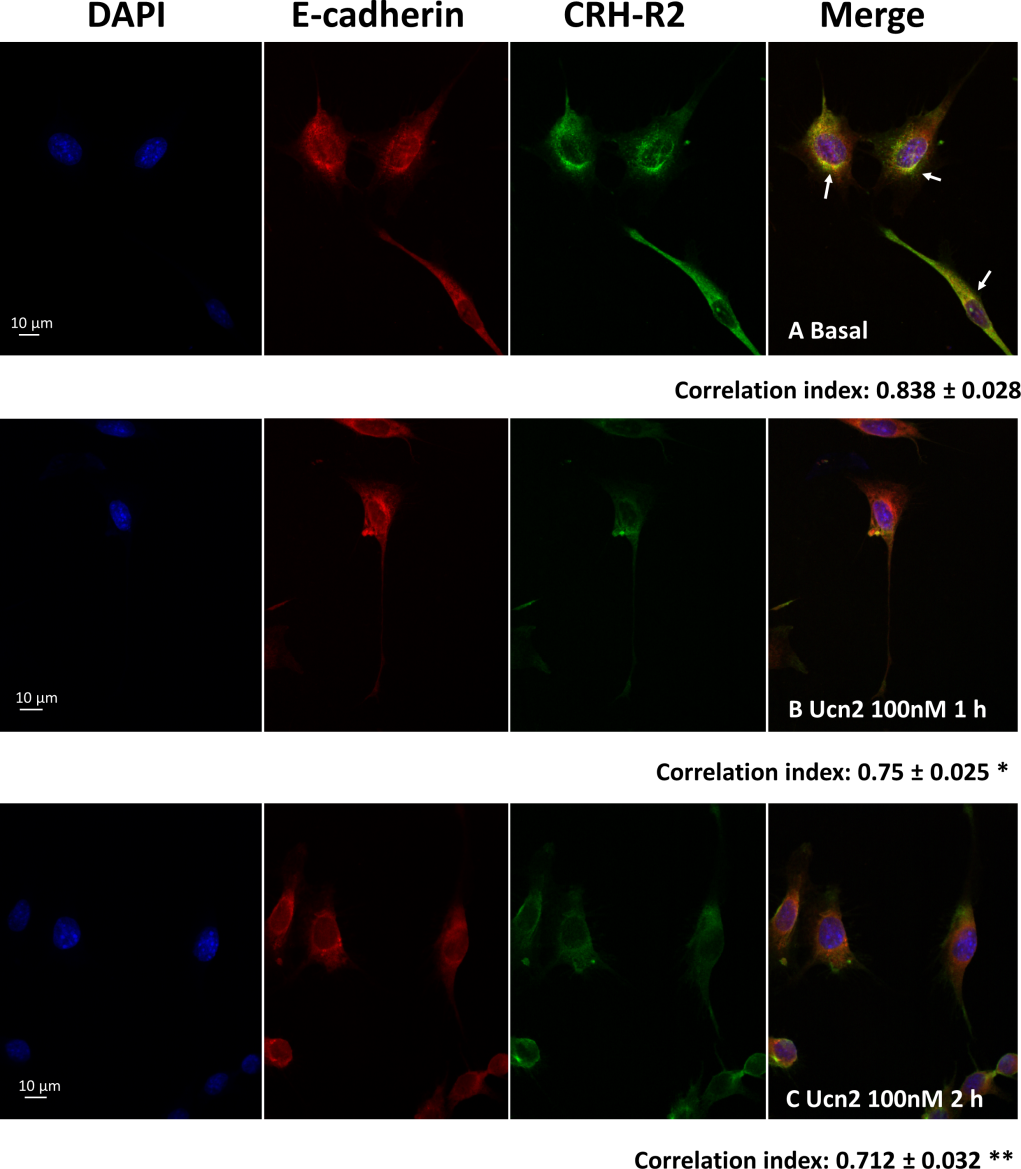
CRH-R2 and E-cadherin membrane distribution in mHypoA-2/30 cells. **(A)** Basal fluorescent signal of DAPI, CRH-R2 and E-cadherin in hypothalamic cells; **(B)** Changes in CRH-R2 cellular localization after treatment with 100nM of Ucn2 for 1 hour and **(C)** for 2 hours, visualized by fluorescent confocal microscopy. Cell nucleus was stained using DAPI, while double immunofluorescence examined E-cadherin (red) and CRH-R2 (green). Arrows show colocalization between CRH-R2 signal and that of the plasma membrane marker E-cadherin. One way ANOVA showed a significant effect of Ucn2 treatment decreasing colocalization (F_(2,15)_ =4.619 p<0.05). Values are the mean ± SEM of the correlation index of pixels (colocalization) *P< 0.05, **P <0.01 *vs*. basal.

**Figure 2:**
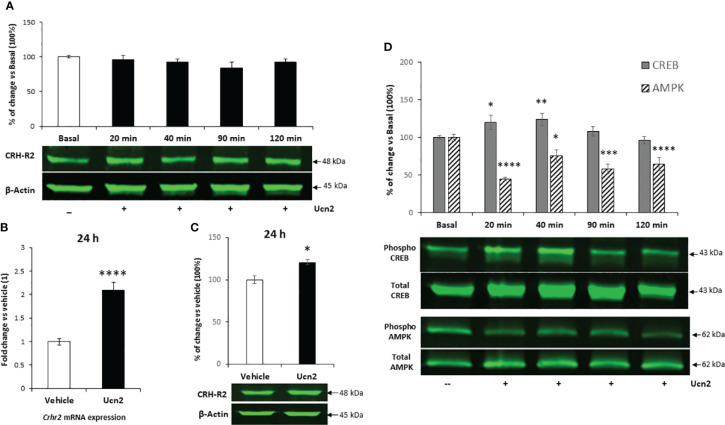
CRH-R2 expression and intracellular signalling in mHypoA-2/30 **(A)** Representation of CRH-R2 total protein level normalized by that of β-Actin in mHypoA2/30 cells evaluated after 20, 40, 90 and 120 min of 100 nM Ucn2 treatment . **(B)** Gene expression of Crhr2 in mHypoA-2/30 cells after 24 hours treatment with water (veh) or Ucn2 (100 nM). Values are the mean ± SEM of the normalized expression of each gene by that of Actb expressed in arbitrary units as fold change vs the expression in water treated cells (considered as 1) (n=8/group) t=-6.342 P< 0.0001. **(C)** CRH-R2 total protein level normalized by that of β-Actin in mHypoA2/30 cells after 24 hours treatment with water (veh) or Ucn2 (100 nM). Values are the mean ± SEM expressed as % of change vs water treated cells (=100%)(n=4/group) t=-3.489 P=0.01. **(D) ** Phospho-CREB and phospho-AMPK levels normalized by those of total CREB and total AMPK in mHypoA2/30 cells treated with 100 nM Ucn2 at different time points, one-way ANOVA showed a significant effect of Ucn2 treatment (F(4,64)=4.177 p<0.01 for CREB), (F(4,63) = 9.712 p<0.0001 for AMPK), n=6-8/group. Values are the mean ± SEM of the fluorescent signal of CRH-R2/β-Actin ratio, pCREB/tCREB ratio or pAMPK/tAMPK expressed as % of basal (=100%). *P< 0.05, **P <0.01 ***P <0.001, ****P <0.0001 vs. veh or basal.

Preliminary experiments in mHypoA-2/30 cells confirmed endogenous expression of CRH-R2 by employing indirect confocal microscopy and immunostaining using an antibody that recognizes the C-terminus of the 7-transmembrane receptor. Moreover, sustained Ucn2 treatment for up to 2 hours reduced intensity of the CRH-R2 fluorescence signal and yellow signal (indicative of CRH-R2 and E-cadherin co-localization) (**Figure 1**) suggesting receptor redistribution towards the cytoplasm and Ucn2-induced receptor internalization. Immunoblotting of whole cell lysates with a N-terminus-specific CRH-R2 antibody, identified a major protein band of around 48 kDa, confirming CRH-R2 endogenous expression in mHypoA-2/30 cells. Protein levels remained unaltered upon treatment with Ucn2 (100nM) for up to 120 min (**Figure 2A**), suggesting that short-term exposure of these cells to Ucn2 does not modify total CRH-R2 expression levels. Interestingly, longer exposure of mHypoA-2/30 cells to Ucn2 (100nM) for 24 h, significantly increased *Crh-r2* gene mRNA levels by 2x fold (**Figure 2B**) and protein expression by 20% (**Figure 2C**), suggesting a positive feedback loop of Ucn2 regulating CRH-R2 levels.

Investigation of receptor functional characteristics, showed a transient increase on CREB phosphorylation at Ser-133 after treatment with Ucn2 (100nM) for 20 and 40 min in mHypoA-2/30 cells, returning to basal levels at 120 min (**Figure 2D**). Interestingly, during the same treatment period we observed a sustained inhibitory effect of Ucn2 on AMPK activation that reduced phospho-Thr172 levels by 40-60% (**Figure 2D**).

**Figure 3:**
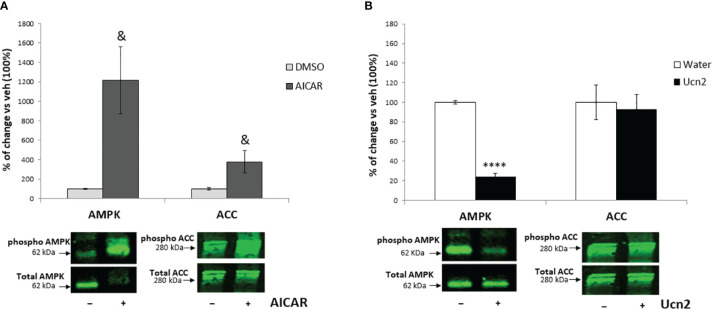
Comparison of AMPK and ACC phosphorylation in hypothalamic cells after treatment with AICAR or Ucn2. Phosphorylation of AMPK and its target protein ACC after 2 hours treatment with AICAR **(A)** or Ucn2 **(B)** vs its vehicles, DMSO and water respectively (n=4-7/group). Significant effects of AICAR on AMPK phosphorylation: t=-2.737 P<0.05; ACC phosphorylation: t=-2.422 P<0.05 and of Ucn2 on AMPK phosphorylation: t=20.487 P< 0.0001 were observed. Values are the mean ± SEM of the fluorescent signal of pAMPK/tAMPK or pACC/tACC ratio and are expressed as % of vehicle values (100%); ****P< 0.0001 vs. water, & P<0.05 vs DMSO.

Since the AMPK-ACC pathway plays a key role in hypothalamic control of feeding, we investigated its signalling characteristics in mHypoA-2/30 cells. Our preliminary results indicated a functional signalling pathway since treatment of cells with the AMPK activator AICAR (1mM for 120 min) induced significant phosphorylation of AMPK by 10-fold and a downstream increase of ACC inactivating phosphorylation at Ser-79 by 4-fold (**Figure 3A**). However, despite inhibition of phospho-AMPK levels by almost 80%, Ucn2 did not alter basal ACC phosphorylation (**Figure 3B**), possibly uncoupling Ucn2 signalling downstream of AMPK from ACC regulation.

### Regulation of hypothalamic genes involved in feeding by Ucn2 in mHypo-2/30 cells

**Figure 4:**
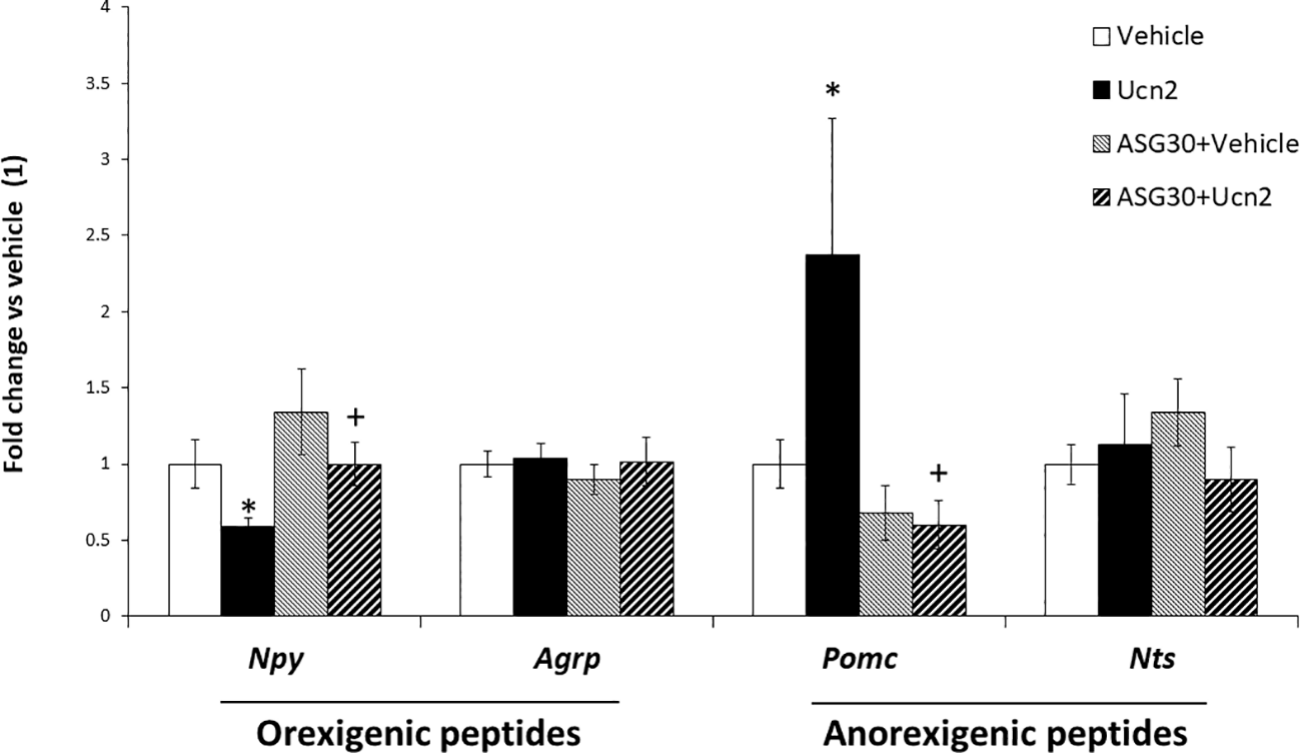
CRH-R2 modulation of appetite regulatory genes. Gene expression of feeding related peptides in mHypoA-2/30 cells treated with ASG30 (1µM) and Ucn2 (100 nM). Two way ANOVA showed a significant effect of ASG30 pre-treatment (F(1,23) =4.816 p<0.05) and of Ucn2 treatment (F(1,23) =4.825 p<0.05) on Npy expression; and a significant effect of ASG30 pre-treatment (F(1,28) =9.995 p<0.01) and interaction between ASG30 pre-treatment and Ucn2 treatment (F(1,28) =4.797 p<0.05) on Pomc expression. Values are the mean ± SEM of the normalized expression of each gene by that of Actb expressed in arbitrary units as fold change in the expression in cells with vehicle (=1) (n=5-14/group). *P< 0.05 vs vehicle, +P< 0.05 vs Ucn2.

In agreement with its anorexigenic effect *in vivo*, Ucn2 treatment decreased by 40% gene expression of the orexigenic peptide *Npy*, whereas a 2.5 x fold increase in *Pomc* mRNA levels (**Figure 4**) was observed. In contrast, *Agrp* and *Nts* mRNA levels were not affected. To confirm that Ucn2 actions were mediated via CRH-R2, we used the specific CRH-R2 antagonist antisauvagine 30 (ASG30); when cells were pre-treated with ASG30, Ucn2 effects on *Npy* and *Pomc* mRNA expression were abolished (**Figure 4**).

### CRH-R2 internalization and impact on Ucn2 regulation of hypothalamic genes involved in feeding

**Figure 5:**
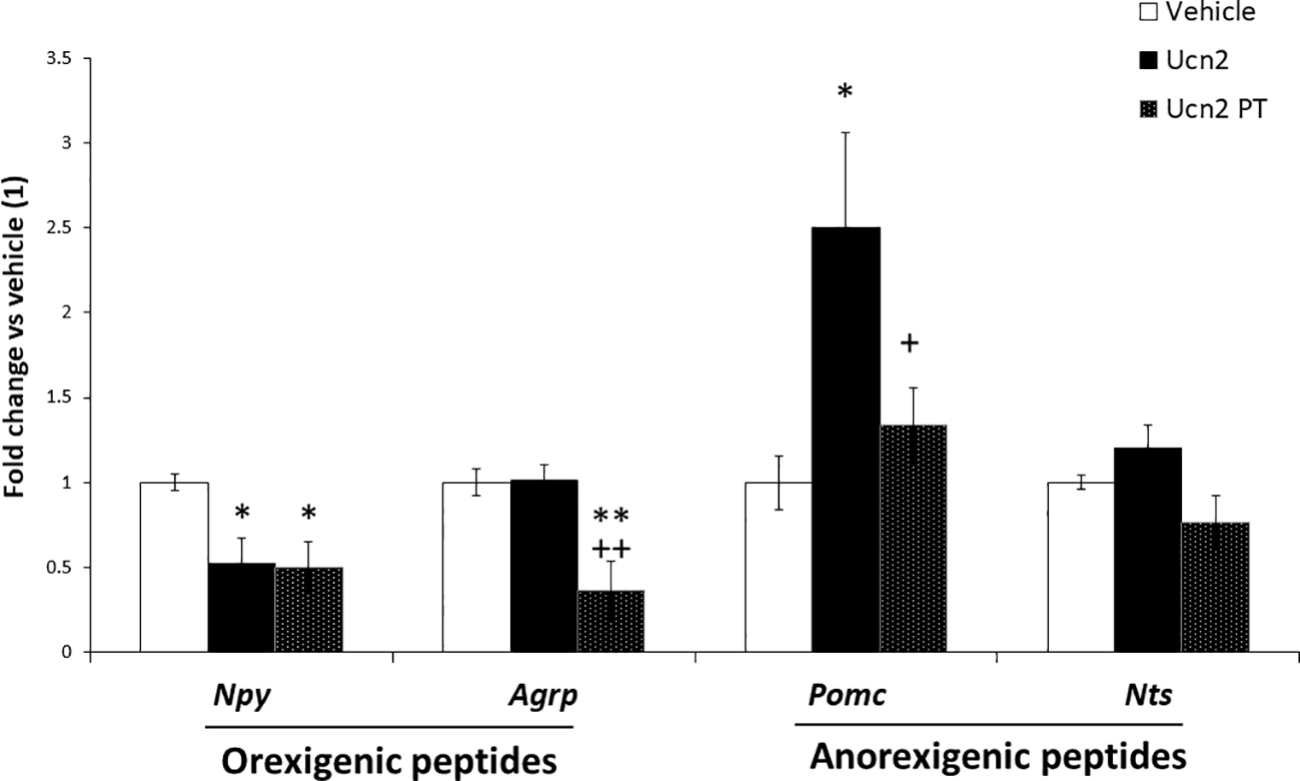
Effects of Ucn2-induced CRH-R2 internalization on appetite regulatory gene expression. Gene expression of feeding related peptides in mHypoA-2/30 cells treated with Ucn2 (100nM) for 24 hours or with the same dose twice (Pre-treatment, Ucn2 PT), a 1 hour Ucn-2 pre-treatment was done in order to promote CRH-R2 internalization and then the second stimulation lasted 24 hours. One way ANOVA showed a significant effect of treatment on Npy expression (F(2,13) =5.622 p<0.05); Agrp expression (F(2,17) =9.773 p<0.01) and Pomc expression (F(2,15) =4.743 p<0.05). Values are the mean ± SEM of the normalized expression of each gene by that of Actb expressed in arbitrary units of the fold change vs the expression in vehicle treated cells (considered as 1) (n=5-8/group). *P< 0.05 **P<0.01 vs Vehicle, +P<0.05 ++P<0.01 vs Ucn2.

To characterise the impact of CRH-R2 internalization on cellular responsiveness to Ucn2, we determined Ucn2 effects on regulation of key hypothalamic genes following a single pre-treatment step with Ucn2 for 1 hour, which was shown to induce significant CRH-R2 subcellular redistribution (**Figure 1**). Interestingly, we observed distinct Ucn2 responses following receptor internalization; for example, Ucn´s 2 inhibitory effect on *Npy* expression was independent of a Ucn2 pre-treatment step that induced receptor internalization, whereas stimulation of *Pomc* expression was abolished. Surprisingly, induction of CRH-R2 internalization also led to reduction of *Agrp* mRNA levels (**Figure 5**), an effect not observed in cells that were not exposed to Ucn2 pre-treatment.

### Ucn2 actions on the circadian clock in mHypoA-2/30 cells

We analysed the expression pattern of bona fide core clock genes (*Clock*, *Cry1, Cry2* and *Bmal1*) in mHypoA-2/30 cells. *Clock* and *Bmal1* act as transcriptional activators in an autoregulatory network of 24 hours; they positively regulate the expression of *Cry1* and *Cry2* and the period genes *Per1* and *Per2.* The proteins CRY1, CRY2, PER1 and PER2 dimerize, producing a complex that translocate into the nucleus where they interact with CLOCK and BMAL1, repressing their own transcription (28).

**Figure 6:**
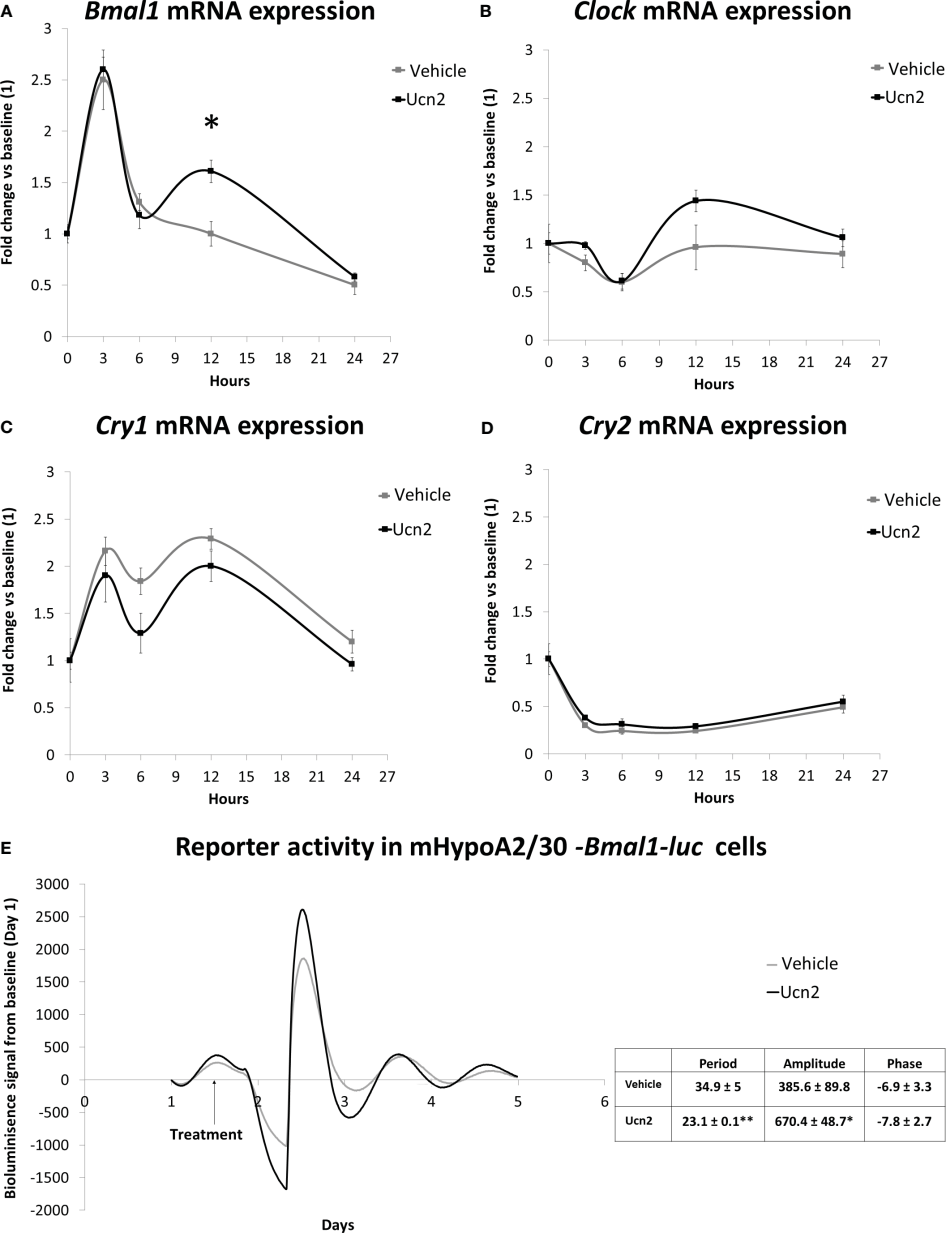
CRH-R2 modulation of circadian genes. Gene expression of circadian regulatory transcription factors in mHypoA-2/30 cells treated with water or Ucn2 (100 nM) at 3, 6, 12 and 24 hours. **(A)** *Bmal1* gene expression at 12 hours, t=-3.274 P<0.05. AUC for water: 32.39, for Ucn2: 41.22. **(B)** *Clock* gene expression AUC for water: 20.58, for Ucn2: 26.51. **(C)** *Cry1* gene expression. AUC for water: 44.07, for Ucn2: 36.77. **(D)** *Cry2* gene expression. AUC for water: 8.58, for Ucn2: 9.95. Values are the mean ± SEM of the normalized expression of each gene by that of *Tbp* expressed in arbitrary units of fold change *vs* the expression in vehicle treated cells (considered as 1)(n=4/group). **(E)** Average real time bioluminescence signal of mHypoA2/30 *–Bmal1-luc* reporter cells. Cells were treated with water or 100nM Ucn2 at 36h after dexamethasone synchronization. Figure represents photon counts/sec against time (days) in 4-8/treatment. *P< 0.05, **P< 0.01 *vs* vehicle.

Interestingly, incubation with 100 nM Ucn2 for 12 hours after synchronization changed the circadian pattern of expression of *Bmal1*. Ucn2 treated cells exhibited significantly increased *Bmal1* mRNA levels (**Figure 6A**) with a biphasic increase in *Bmal1 levels* with the second weaker peak observed around 12h. Although *Bmal1* is a transcriptional activator of *Cry* genes, the higher *Bmal1* expression was not associated with any changes in the *Cry1 or Cry2* mRNA temporal expression pattern (**Figures 6C, D**) suggesting requirements for additional components such as *Clock* which was not affected by Ucn2 treatment (**Figure 6B**).

We confirmed that mHypoA-2/30 cells exhibit circadian oscillations in our *Bmal1-luc* expressing circadian reporter line. We identified an oscillation pattern of *Bmal1-luc* bioluminescence in mHypoA-2/30 cells with a period of 34.9 ± 5 hours after synchronisation with dexamethasone, a more robust synchronizer of clock genes compared to FBS shock (29, 30). Given the variable effects of glucocorticoids and serum shock synchronisation methods on circadian gene expression (29), future comparison experiments could reveal changes in the oscillation period found in the mHypoA-2/30 cells. To further assess the impact of Unc2 on the clock, particularly on *Bmal1*, we treated the mHypoA2/30-*Bmal1*-*luc* reporter cells with 100 nM Ucn2 after 36 hours of synchronization and found increased amplitude of luciferase activity by 70%, associated with a concomitant decrease of 30% in the period for Bmal1 rhythmicity (**Figure 6E**).

## Discussion

Recent evidence suggests that chronic stress conditions associated with prolonged CRH-R2 activation might contribute to hyperphagia by development of resistance to the anorexigenic effects of CRH and Ucn2 in animals (3). However, detailed characterisation of the molecular properties of these hypothalamic peptides and downstream pathways is challenging to perform *in vivo* because of the complex structure and heterogeneity of hypothalamic neurons and numerous neuronal phenotypes present in various hypothalamic nuclei, as well as difficulties around use of primary hypothalamic cultures. Adequately characterised hypothalamic cell models like the adult-derived hypothalamic mHypoA-2/30 that lack the complexity and integrated network of neuronal inputs, connections and signalling are emerging as valuable tools for the molecular analysis and characterisation of hypothalamic neuronal function (31). The mHypoA-2/30 cells express endogenous functional CRH-R2 and are suitable for *in vitro* characterization aspects of CRH-R2 signalling. A significant proportion of endogenous CRH-R2 can be detected at or near the plasma membrane, a subcellular localization pattern similar to other various cell lines (32, 33). Prolonged agonist treatment induced internalization of CRH-R2, an event that potentially alters mHypoA-2/30 cellular responsiveness to Ucn2. Our results also identified a parallel up-regulation of *Crh-r2* mRNA and protein levels after prolonged exposure of cells to Ucn2. This positive-feedback response to Ucn2 in mHypoA-2/30 cells is supported by previous observations of increased *Crh-r2* expression in the hypothalamic paraventricular nucleus (PVN) of early-life stressed adult rats, a stressor that maintains the *Ucn2* mRNA content increased in this region (3). In addition, Ucn2 stimulatory effect on *Crh-r2* gene expression has also been reported in pituitary gonadotropic tumour mouse LβT2 cells (34), modulating the endocrine interaction between the adrenal and gonadal axis. A similar response has been observed in receptors of other hypothalamic peptides involved in feeding behaviour like the leptin receptor (ObRb) in hypothalamic cells (35) as well as in animal models of obesity in which, for example, increased circulating leptin levels with higher gene expression of hypothalamic ObRb suggest desensitization of its functionality as the expression of downstream feeding peptides is also altered (36, 37). Therefore, increased *Crh-r2* expression by prolonged exposure to Ucn2 could represent a compensatory mechanism of a homologous desensitization process although further studies are required to establish the biological mechanism.

We observed here that in mHypoA-2/30 cells the CRH-R2 receptors are functionally coupled to phosphorylation of downstream targets that lead to CREB activation and inhibition of AMPK activity. Ucn2 did not affect levels of the inactivating phosphorylation at Ser-79 of ACC, the downstream target of AMPK, suggesting an uncoupling signalling of AMPK on ACC phosphorylation. However, inhibition of AMPK by Ucn2 might allow other post-translational modifications to occur such as amino-acid-induced allosteric activation of ACC (38; 39) enabling its signalling. Future experiments focusing on the characteristics of Ucn2 modulation of ACC activity will delineate this pathway in the central nervous system as part of energy homeostasis under stress conditions.

Both CREB and AMPK pathways differentially regulate expression of orexigenic and anorexigenic hypothalamic peptides. In the mHypoA-2/30 cell line Ucn2 regulation of this kinase network, might link stress signals to the transcriptional control of peptides involved in feeding regulation such as *Npy* and *Pomc* genes (15, 40) as we observed an enhanced expression of anorexigenic genes and inhibition of orexigenic signals, similarly to effects of insulin or leptin on hypothalamic peptide expression (13).

In the mHypoA-2/30 cellular model, CRH-R2 agonist-induced receptor internalization differentially influenced Ucn2 effect on transcription of anorectic and orexigenic peptides suggesting distinct pathways. Following receptor internalisation, Ucn2 potency on *Pomc* expression up-regulation is diminished; a similar uncoupling of POMC hypothalamic neurons from hormonal regulation have been reported in studies investigating hypothalamic action of hormones like insulin (41) or leptin (42). Although these studies are focused on POMC-expressing neurons of the hypothalamus and its role in the regulation of food intake by maintaining a melanocortin-dependent anorexigenic tone; it is possible that similar pathways also regulate other POMC-actions downstream of CRH activation of the hypothalamus-pituitary-adrenal (HPA) axis controlling the stress response. Previous studies in chronically stressed animals demonstrated that desensitization of the CRH receptors could lead to decrease in POMC synthesis and possibly an attenuated response of the HPA axis to stressful stimulus (43).

Interestingly, in mHypoA-2/30 cells, not all Ucn2 actions are dampened following receptor internalisation; in conditions of enhanced CRH-R2 receptor internalisation, Ucn2 signalling is directed towards downregulation of mRNA expression of *Agrp*, potentially contributing to inhibition of orexigenic signals. This finding is consistent with the view that internalised GPCRs could influence the activation of alternative signalling pathways inducing outcomes that sometimes differ from ‘canonical’ plasma membrane receptor signalling (6). Additionally, emerging evidence shows the recruitment of alternative G alpha subunits (Gi and Go) together with β-arrestin as part of the desensitization process of CRH-R2 by Ucn2, with a possible impact on downstream signalling pathway activation (44). Therefore, our results suggest a potential change in hypothalamic CRH-R2 signalling on both anorexigenic and orexigenic gene expression after the agonist-induced internalization of the receptor.

Very little is known about the regulation of circadian genes by hypothalamic CRH-R2 although functional interactions have been suggested since CRH-R2 ‘global’ or site-specific silencing from the ventromedial hypothalamus alters food intake patterns during the dark-light cycle (23, 45). Also, i.c.v. injection of Ucn2 in rats exerts maximal anorexigenic effects at the end of the dark phase (22), suggesting a potential stress-induced modulation of CRH-R2 on circadian feeding regulation. Such action of CRH-R2 might involve increased mRNA levels of *Bmal1* and rhythm amplitude of the circadian regulator *Bmal1* as we observed in the mHypoA-2/30 cells after 12 hours treatment with Ucn2. Bmal1 has shown to be particularly relevant in the regulation of energy metabolism (46) and its gene expression is modulated positively by CREB phosphorylation (47) and negatively by Cry and Per proteins (48), which influence *Bmal1* expression through the cyclic nature of the core clock transcription factors. Although not analysed in the present work, phosphorylation of CREB at Ser 142 regulates the expression of *Per1* with a possible effect on *Bmal1* expression (49). Therefore, Ucn2 could enhance *Bmal1* mRNA levels through phosphorylated CREB-induced intracellular pathways. In a physiological context, the increased amplitude and shortened period of *-Bmal1-luc* oscillation by Ucn2 observed in our *in vitro* experiments might represent a mechanism contributing to its anorexigenic effects, since attenuation in the amplitude of *Bmal1* expression in the suprachiasmatic nucleus and loss of its rhythmicity in the arcuate nucleus is observed in rats fed only during the light phase of the cycle (50). As deletion of *Bmal1* influences the rhythmic expression of all systems including the feeding peptides *Npy*, *Pomc* and *Agrp* in hypothalamic cells (51), the increased amplitude in *Bmal1* expression by Ucn2 could impact the circadian rhythmicity in the expression of these appetite-related molecules possibly potentiating an anorexigenic effect during the activity phase as observed *in vivo* (22).

**Figure 7:**
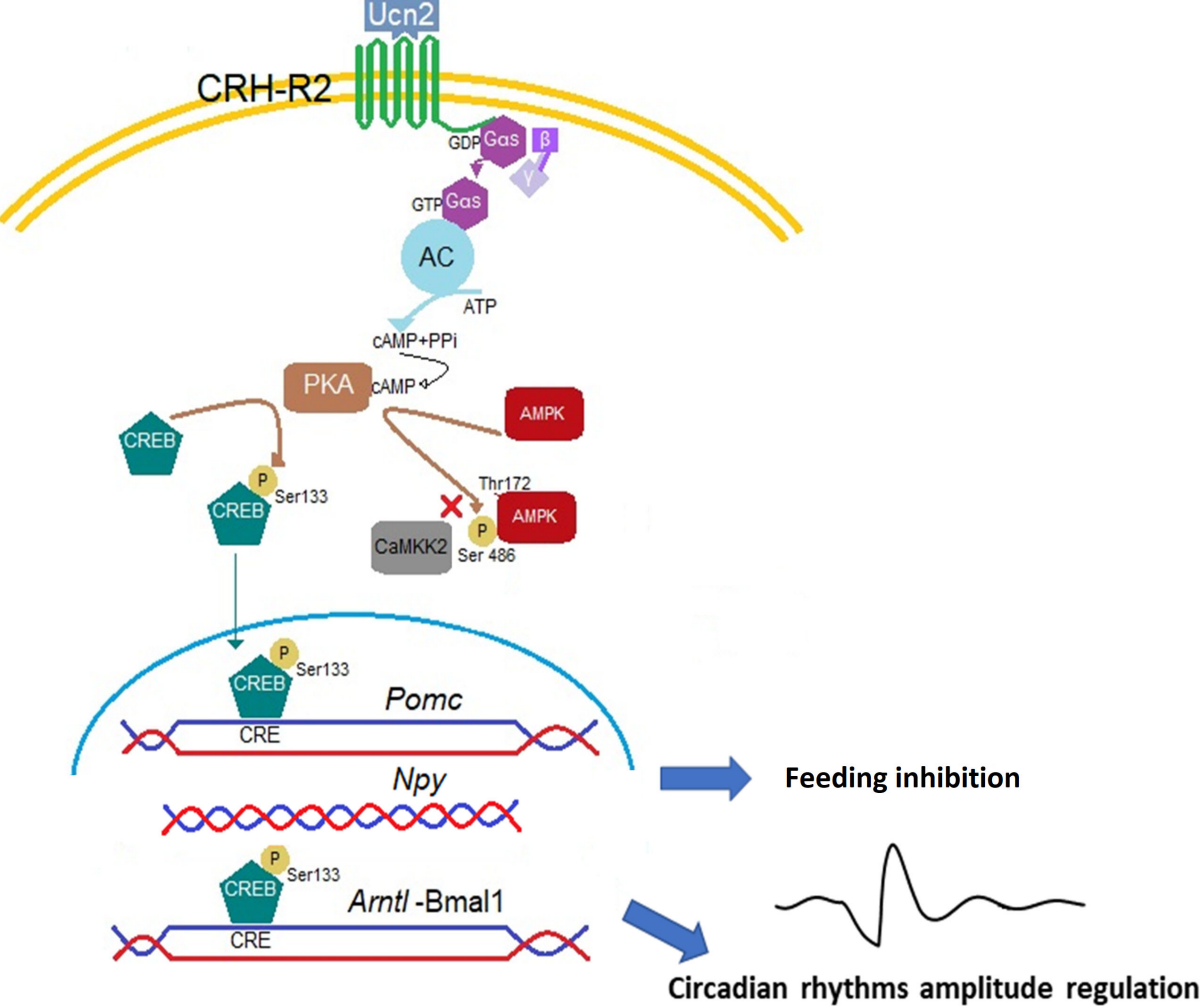
Proposed mechanism of Ucn2 anorexigenic effects on hypothalamic neurons. The binding of Ucn2 to CRH-R2 at the plasma membrane trigger intracellular events such as the activation of adenylyl cyclase (AC) by the α_s_ subunit of the G protein, leading to increases in the concentration of cyclic AMP (cAMP) and activation of PKA which phosphorylates the transcription factor CREB at the Ser133 residue and the enzyme AMPK at the Ser486, blocking its phosphorylation at the activation site Thr172 by CaMKK2, inhibiting AMPK activity; while phospho-CREB translocate to the nucleus and binds CRE sites in the *Pomc* and *Arntl* promoters, increasing gene expression. Repression in *Npy* gene expression could be also driven by binding of CREB on the *Npy* promoter. All these changes promote anorexia particularly detectable at the activity phase of the animal by activation of CRH-R2.

In conclusion, we are providing first experimental data showing that the hypothalamic mHypoA-2/30 neurons endogenously express functional CRH-R2 receptors that regulate activity of the intracellular signals CREB and AMPK and downstream transcription levels of the appetite-related genes *Pomc* and *Npy*, which are likely to respond differently after CRH-R2 ligand-induced internalization. CRH-R2 signalling could be a molecular link between energy metabolism and circadian rhythms via the modulation of hypothalamic *Bmal1* expression. A schematic depiction of the potential mechanisms is presented in **Figure 7**. These results contribute to our understanding of the molecular mechanisms involved in CRH-R2 modulation of the circadian and feeding regulatory genes and in conjunction with *in vivo* models will further describe the central control of appetite during conditions of acute and chronic stress exposure.

## Data availability statement

The raw data supporting the conclusions of this article will be made available by the authors, without undue reservation.

## Ethics statement

Ethical approval was not required for the studies on animals in accordance with the local legislation and institutional requirements because only commercially available established cell lines were used.

## Author contributions

VA-A: Conceptualization, Data curation, Formal Analysis, Investigation, Methodology, Writing – original draft. PdG: Conceptualization, Funding acquisition, Methodology, Resources, Writing – original draft. HL: Conceptualization, Writing – original draft. RD: Data curation, Methodology, Writing – original draft. DG: Conceptualization, Formal Analysis, Funding acquisition, Methodology, Project administration, Resources, Supervision, Writing – original draft.
