# Supplementary figures and images for: CRH-R2 signalling modulates feeding and circadian gene expression in hypothalamic mHypoA-2/30 neurons
Source: Front Endocrinol (Lausanne). 2023 Oct 11;14:1266081. doi: 10.3389/fendo.2023.1266081 (PMC10600019; doi:10.3389/fendo.2023.1266081)

# Appendix

## Complete gels

Figure 2, Panel A

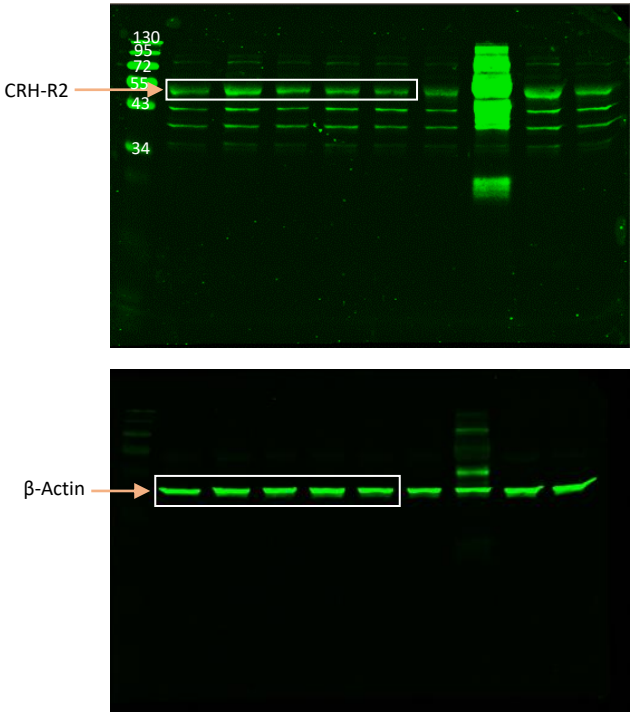

Figure 2, Panel C

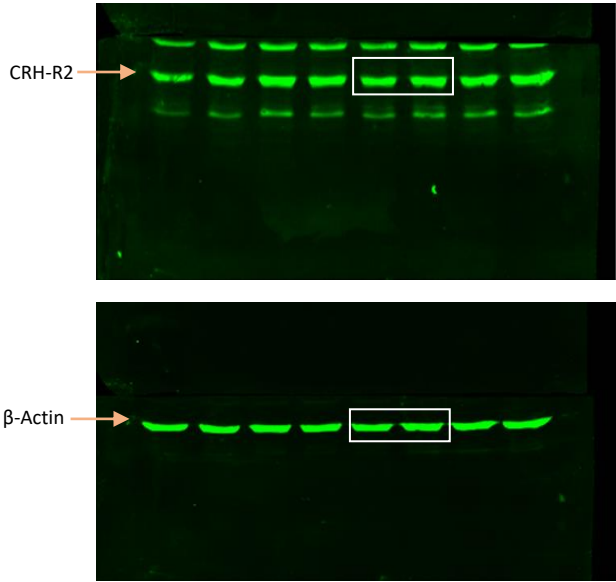

Figure 2, Panel D

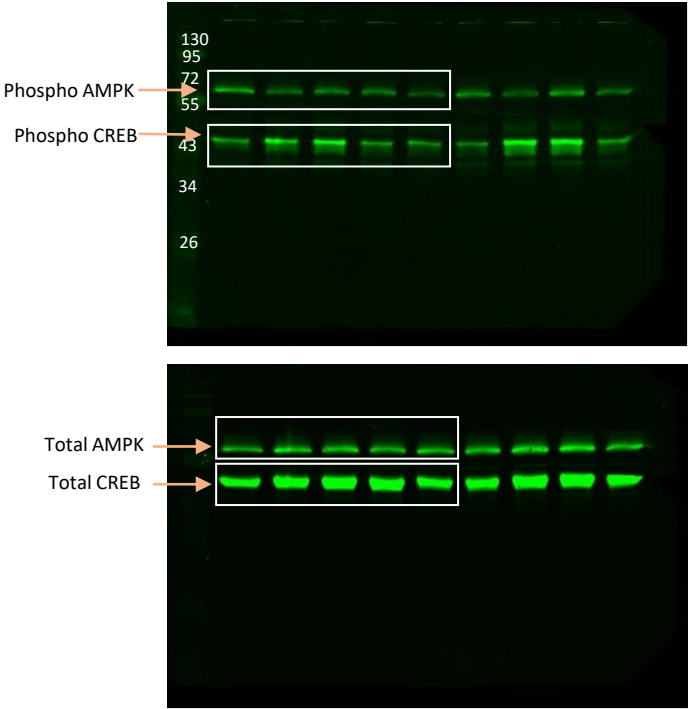

Figure 3

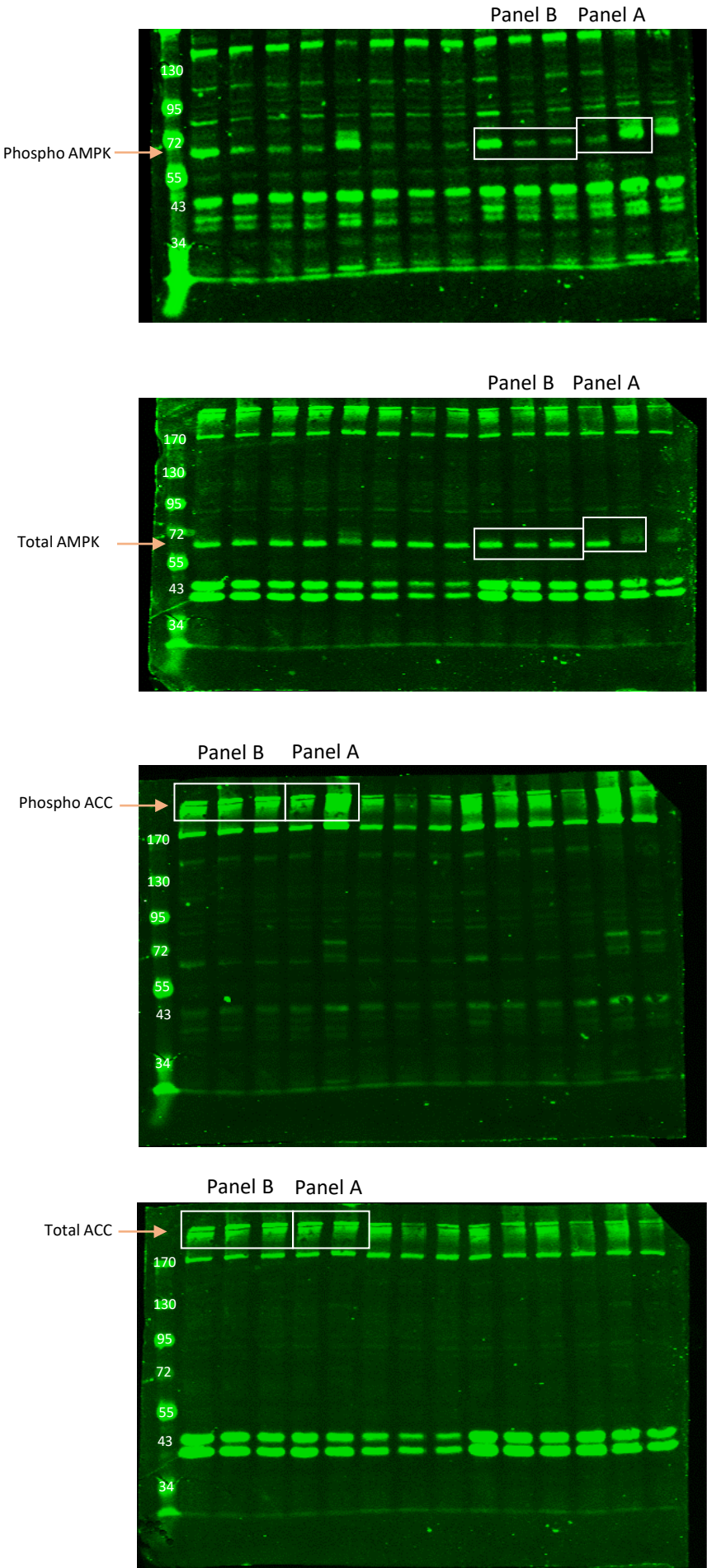

Supplement: Supplementary file 1 [file Image_1.pdf]
